# The association of serum IL-33/ST2 expression with hepatocellular carcinoma

**DOI:** 10.1186/s12885-023-11179-5

**Published:** 2023-07-28

**Authors:** Xiaolan Pan, Jinfeng Liu, Meiqin Li, Yihua Liang, Zhimin Liu, Ming Lao, Min Fang

**Affiliations:** Department of Clinical Laboratory, Guangxi Medical University Cancer Hospital, Nanning, 530021 Guangxi China

**Keywords:** IL-33, ST2, Hepatocellular carcinoma, Prognosis, Meta, Cancer risk

## Abstract

**Background:**

IL-33 is a multifunctional cytokine with dual functions. However, the clinicopathological and prognostic significance of IL-33 in cancer patients, especially in patients with hepatocellular carcinoma (HCC), remains controversial. Therefore, we conducted a study of 565 patients with HCC and 561 healthy controls and performed a meta-analysis to quantitatively evaluate the above problems.

**Methods:**

We collected blood from 565 patients with HCC and 561 healthy controls. ELISA was used to detect the concentrations of IL-33 and ST2 in the serum, and RT‒PCR was used to detect the levels of IL-33 and ST2 mRNA. Meanwhile, we collected comprehensive literature on IL-33 and the clinical characteristics of cancer patients retrieved from the PubMed, Web of Science and CNKI databases as of December 2022. An odds ratio (OR) with a 95% confidence interval (CI) was used to estimate the impact through overall and stratified analyses.

**Results:**

Compared with the healthy control group, the levels of ST2 mRNA and serum in the peripheral blood of HCC patients increased (*p* < 0.05), while the levels of IL-33 mRNA and serum showed no significant difference between the two groups (*p* > 0.05). In the meta-analysis section, at the tissue level, the overall analysis showed that the expression of IL-33 was positively correlated with tumor stage, histological grade, distant metastasis, and tumor size. Compared with patients with low IL-33 expression, the 3-year overall survival (OS) rate (OR = 3.467, *p* < 0.001) and 5-year OS rate (OR = 2.784, *p* < 0.001) of patients with high IL-33 expression were lower. At the serum expression level, the overall analysis showed that the expression of IL-33 increased the risk of cancer, and the serum level of IL-33 was positively correlated with tumor stage and vascular invasion.

**Conclusion:**

IL-33/ST2 is a useful predictive or prognostic biomarker in clinical evaluation and may be used as a potential therapeutic target, but much research is needed to verify this hypothesis.

## Introduction

The interleukin-33 (IL-33) gene is located on human chromosome 9 (9q24. 1), and its encoded IL-33 precursor (ProIL-33) is in the nucleus. Il-33 is widely expressed in cells involved in the formation of the body's defense system, such as epithelial cells and endothelial cells. The IL-33 receptor ST2, a member of the Toll-like receptor (TLR)/IL-1 receptor family, is located on human chromosome 2 [1]. ST2 is expressed in a variety of cells, including epithelial cells, fibroblasts, and immune cells. The inflammatory environment may be involved in the occurrence and development of tumors by mediating the release of IL-33 and via the IL-33/ST2 signaling pathway. Many reports have shown that IL-33 is highly expressed in a variety of chronic inflammatory conditions, such as chronic hepatitis B, liver fibrosis, and precancerous lesions, suggesting that IL-33 may play an important role in the progression of inflammation to tumors [2–6].

Hepatocellular carcinoma (HCC) is a primary tumor of the liver and constitutes more than 90% of the primary tumor of the liver. Hepatocellular carcinoma occurs in approximately 85% of patients diagnosed with cirrhosis [7]. HCC is now the fifth most common cause of cancer worldwide in men [7]. The serum level of IL-33 in HCC patients is higher than that in the control group [8], but whether the local IL-33 expression level of HCC tissue is different from that of normal tissue is still controversial [8–10]. Serum IL-33 levels in patients with advanced HCC [10] and metastatic HCC were significantly higher than those in patients with early-stage HCC or lung metastatic HCC [8, 10], suggesting that there is a significant relationship between systemically distributed IL-33 and disease progression [10]. However, ST2 is believed to block IL-33 from functioning as a cytokine, suggesting that IL-33/ST2 may play a role in inhibiting HCC development [10, 11]. Only by collecting and analyzing more patient samples will it be possible to resolve the controversy over the level of IL-33 expression in HCC patients and its trend with disease progression. Therefore, this study collected 565 patients with HCC and 561 healthy controls for serum level and mRNA level analysis, aiming to supplement more research data to reveal the role of IL-33/ST2 in HCC.

In recent years, increasing attention has been given to the role of IL-33/ST2 in tumorigenesis and development, but the effect and mechanism of IL-33/ST2 on tumor progression are still controversial [12, 13]. In different types of tumors, the effect of IL-33/ST2 expression levels on tumor progression showed obvious inconsistencies [13]. Some believe that the expression level of IL-33/ST2 is elevated in most tumors (both serum and tumor tissue) and is positively correlated with tumor progression [8, 14–17]. Another study suggested that there is also a decrease in the expression level of IL-33/ST2 in tumors (including serum and tumor tissue), and it is negatively correlated with tumor progression [18–21]. Therefore, we conducted a meta-analysis to supplement more research data to reveal the role of IL-33/ST2 in HCC.

## Materials and methods

### Study population

A total of 565 patients with untreated HCC who were admitted to the Guangxi Medical University Cancer Hospital from September 2016 to December 2018 and confirmed by surgical resection or biopsy histopathology were selected. The 561 control subjects were healthy people who were recruited in the same hospital from December 2018 to April 2019. The inclusion criteria of the control group were as follows [22]: (1) No family history of liver cancer or other malignant tumors. (2) No other major diseases, such as serious cardiovascular and cerebrovascular diseases. (3) Absence of tumor suggestion in imaging examination. (4) Normal laboratory results, including inflammatory and immune indicators. (5) Age difference ≤ 5 years between healthy individuals and average age of HCC patients for proper matching. After signing the written informed consent form, each participant was interviewed using the prescribed questionnaire to collect historical information on environmental exposure. For each participant,1.5 ml of EDTA anticoagulant whole blood and 0.5 mL of serum of the participants were collected and stored at -80℃ until analysis. Meanwhile, relevant data (gender, age, drinking status, smoking status, tumor stage, metastasis, and related biochemical indicators) were collected. The study was approved by the Institutional Ethics Committee of Guangxi Medical University Cancer Hospital.

### Detection of serum levels of IL-33 and ST2

Serum IL-33 and ST2 were detected by enzyme-linked immunosorbent assay (ELISA), and the serum concentrations of IL-33 and ST2 were repeatedly determined using a commercial kit Human ST2/IL-33R DuoSet ELISA (R&D Systems; Minneapolis, MN, USA). All operations were strictly performed in accordance with the instructions.

### Quantitative real-time PCR analysis and RNA extraction

mRNA was extracted from peripheral blood cells with TRIzol reagent (Invitrogen). The reverse transcription reaction was performed according to the instructions of the TAKARA reverse transcription kit. RT‒PCR was performed on a PCR amplification instrument (Thermo, USA) using the TAKARA SYBR®Premix Ex Taq™II kit. Briefly, according to the Prime Script RT Reagent Kit with gDNA Eraser kit (TaKaRa) instructions, 1 μL of RNA solution was taken, and its concentration was measured by a Thermo instrument. Immediately, reverse transcription was performed to transcribe mRNA into cDNA. Subsequently, according to the SYBR Premix Ex Taq II kit (TaKaRa) instructions, RT‒PCR was performed in the presence of cDNA and primers. In addition, an internal reference for β-actin was designed, and quality control was carried out throughout the experiment. A comparative 2^−ΔCt^ method was used to calculate the relative IL-33 and ST2 mRNA expression. Primer sequences are as follows: primers for IL-33 amplification were 5′-ATCCCAACAGAAGGCCAAAG-3′ (forward) and 5′-CCAAAGGCAAAGCA CTCCAC-3′ (reverse). The primers for ST2 amplification were 5′-GGATTGAGGCCACTCTGCTC-3′ (forward) and 5′-CCGCCTGCTCTTTCGTATGT-3′ (reverse), and the primers for β-actin amplification (internal control) were 5′-TTGCCGACAGGATGCAGAA-3′ (forward) and 5′-GCCGATCCACACGGAGTACT-3′ (reverse).

### Literature search strategy

To confirm the research, we conducted a literature search using PubMed, Web of Science and CNKI databases in December 2022. The following search terms were used: ("interleukin-33"or "IL-33"or "suppression of tumorigenicity 2 receptor" or "ST2" or "interleukin-1 receptor-like 1" or "IL1RL1") and ("tumor" or "cancer" or " malignancy" or "carcinoma" or" neoplasm"). Unfortunately, few studies have described the relationship between serum and tissue levels of ST2 and tumors. Therefore, the association between ST2 levels and tumors was not included in this meta-analysis.

### Study selection criteria

For the study of IL-33 tissue level, a qualified study was met [23]: (1) cancer research; (2) research on the expression of IL-33 protein on the patient's cancer tissue through immunohistochemical detection methods; (3) sufficient public data; and (4) published full-text articles in English or Chinese. Studies on serum IL-33 expression levels were defined as eligible when they met the following criteria [24]: (1) controlled pathologic studies; (2) detection of serum IL-33 expression in cancer patients by methods such as ELISA; (3) adequate public data; and (4) full-text articles in English or Chinese. When multiple articles in the same group were based on similar patients and used the same test, only the most important articles with the latest or most information were included in the meta-analysis.

### Data extraction

For each qualifying study, we extracted the following data according to standard protocols: first author's name, year of publication, country of origin, name of antibody used, and survival analysis. We primarily elucidated the relationship between IL-33 tissue level and clinicopathological parameters, including tumor stage, distant metastasis, histological grade, lymphatic metastasis, vascular invasion, tumor size and serum level. Importantly, we also studied the relationship between IL-33 tissue levels and OS. Human resources were directly extracted and synthesized from multivariate analysis. For the Kaplan‒Meier curve research, we used the software GetData Graph Digitizer 2.24 (http://getdata-graphdigitizer.com/) to directly digitize and extract the data of the 5-year OS rate and 3-year OS rate.

### Statistical analysis

In this study, for the IL-33 tissue level, the correlation strength between IL-33 tissue level and clinicopathological characteristics or 5-year OS rate and 3-year OS were evaluated by OR and 95% CI. OR > 1 indicated that tumors overexpressing IL-33 had a higher probability of progression and a worse prognosis. In addition, a stratified analysis based on the patient's area and the antibodies used in the study was carried out to explore the potential sources of heterogeneity. For IL-33 serum expression, serum IL-33 levels were extracted as the mean ± standardized difference (SD) in each study, and the standardized mean difference (SMD) was used to estimate the effect size when the mean level differences were significant across studies or different units were used. In the process of data collection, the chi-square-based Q test was used for statistical heterogeneity analysis. The I^2^ value indicated the degree of heterogeneity. w *p* value < 10 and/or I^2^ > 50% was considered significant heterogeneity, and then the random effects model was used [25]; otherwise, the fixed effects model was used [26]. Sensitivity analysis was used to assess the stability of the results, and a funnel plot and Egger’s linear regression test were used to estimate potential publication bias. When the funnel chart was visually symmetric and the *p* value of Egger's test was > 0.05, there was no statistically significant publication bias. Differences in demographic characteristics between cases and controls were compared using Student’s t test for continuous variables and χ^2^ test for categorical variables. Serum levels of IL-33 and ST2 in HCC patients and controls were analyzed using the Mann‒Whitney U test. In this study, STATA software version 14.0 and SPSS software version 24.0 were used for statistical analysis, and GraphPad Prism 8.0.2 was used to draw histograms. All statistical tests were two-tailed, and a *p* value < 0.05 was considered statistically significant.

## Results

### Characteristics of the study subjects

A total of 1126 subjects were included in this study, including 565 cases in the HCC group and 561 cases in the healthy control group. Table 1 lists the relevant clinical information. The average age of HCC patients was 53.62 years old, ranging from 10–89 years old, and the average age of the control group was 52.15 years old, ranging from 22–78 years old. Most patients were male, accounting for 86.19% of the study group. The age, sex, smoking history, and drinking history of HCC patients were not significantly different from those of the control group (*p* < 0.05).

**Table 1:** General characteristics of HCC patients and normal controls

Characteristics	Cases (*n* = 565)	Controls(*n* = 561)	*p*-value
Age(year)			0.546
range	10–89	22–78	
mean	53.62	52.15	
< 40	95	102	
≥ 40	470	459	
Gender			0.453
male	487	488	
female	78	73	
BMI (kg/m2)			0.406
< 18.5	62	51	
18.5–23.9	366	359	
≥ 24	137	151	
Smoking status			0.734
No	344	336	
Yes	221	225	
Alcohol drinker			0.113
No	374	396	
Yes	191	165	
BCLC stage			
A + B stage	259		
C + D stage	306		
Metastasis			
No	480		
Yes	85		

### Analysis of the differences in the mRNA and serum expression levels of IL-33 and ST2

**Fig. 1:**
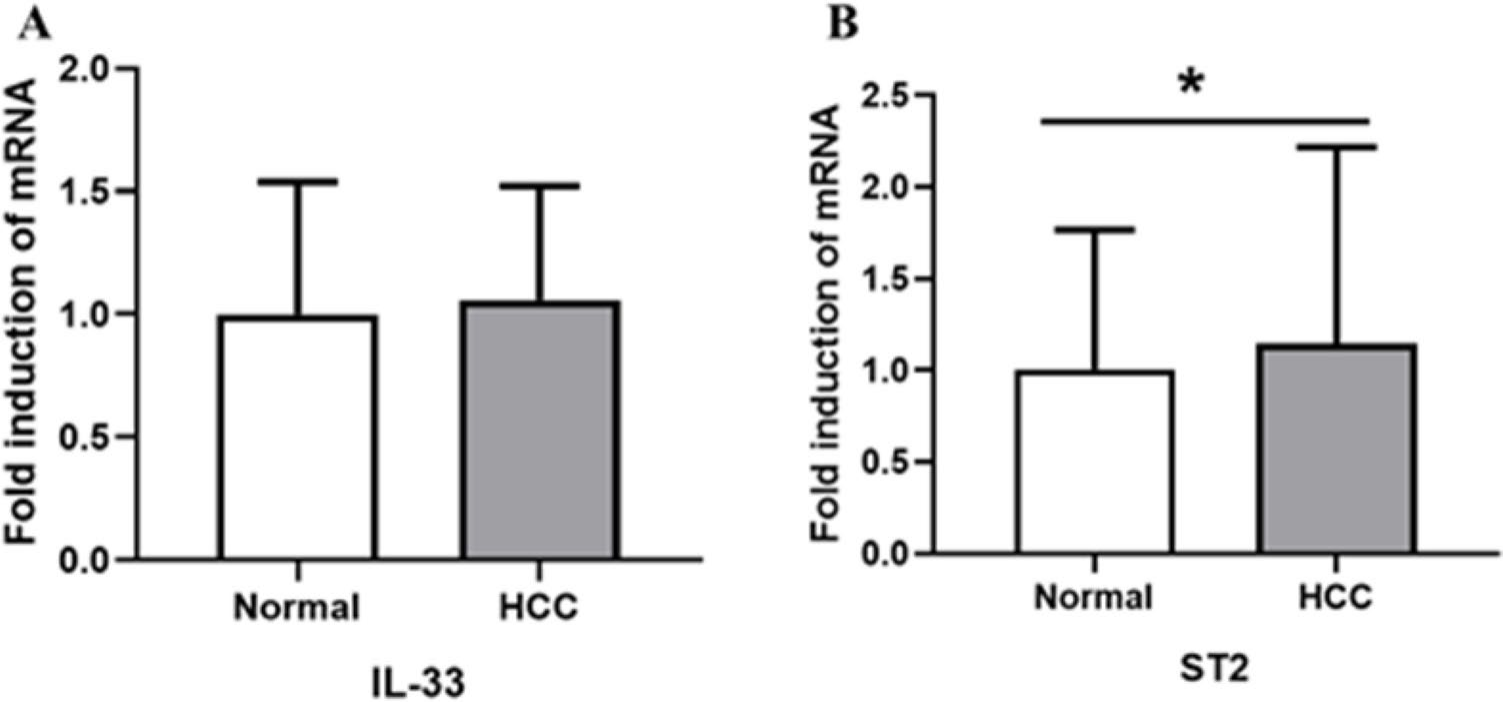
The expression levels of IL-33 (**A**) and ST2 (**B**) mRNA in the HCC group and control group (*p < 0.05)

**Fig. 2:**
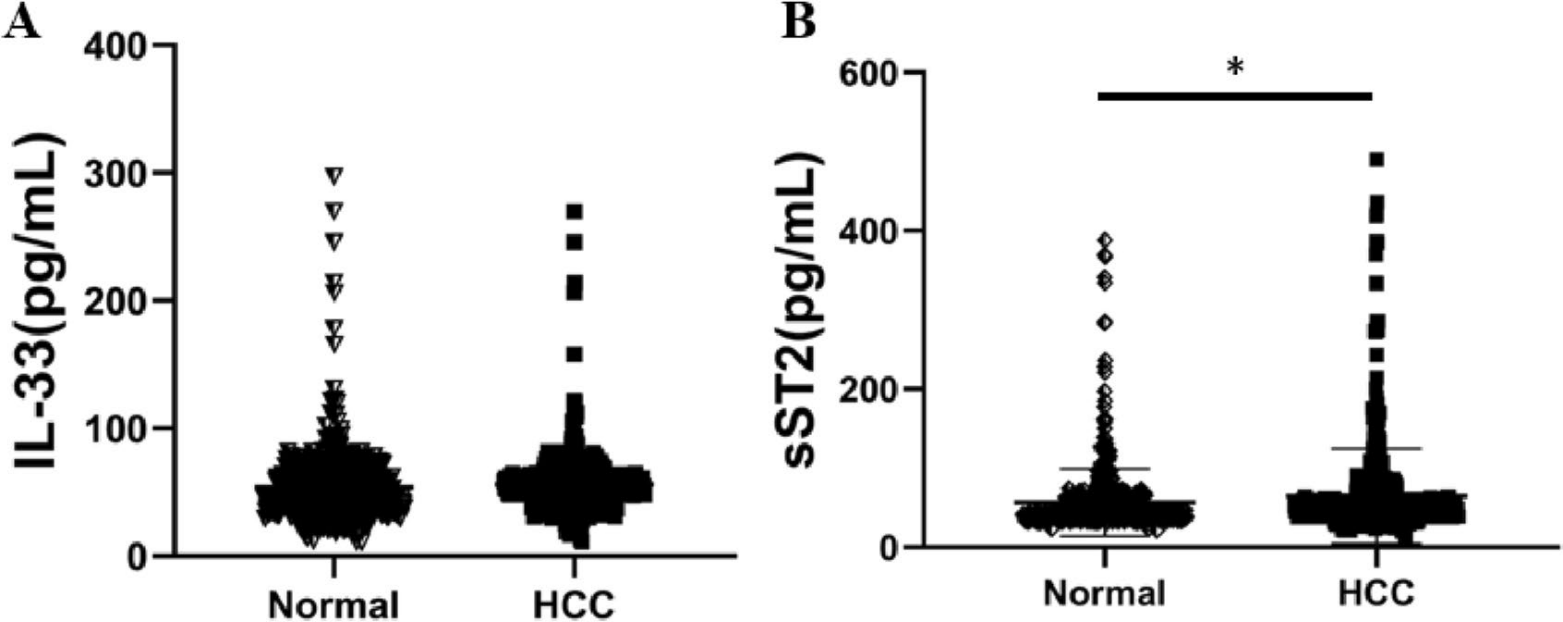
The expression levels of IL-33 (**A**) and ST2 (**B**) serum levels in the HCC group and control group (*p < 0.05)

ELISA and RT‒PCR were used to detect the mRNA and serum levels of IL-33 and ST2 in the HCC group and healthy controls, respectively. The results are shown in Figs. 1 and 2. Compared with the healthy control group, the levels of ST2 mRNA and serum in the peripheral blood of HCC patients increased (*p* < 0.05), while the levels of IL-33 mRNA and serum showed no significant difference between the two groups (*p* > 0.05).

### Characteristics of eligible literature

**Fig. 3:**
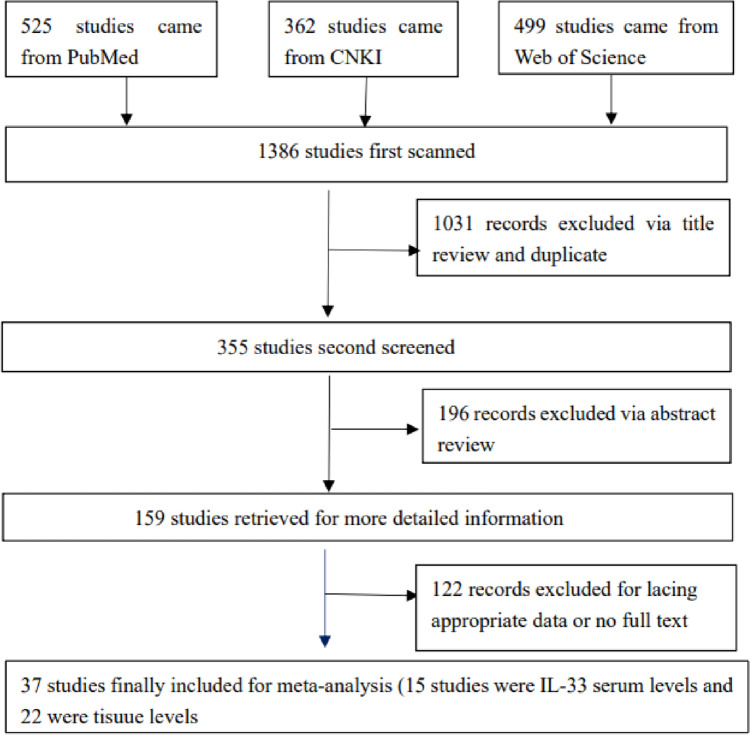
Flowchart of the study selection process prepared for meta-analysis

According to the selection criteria, a total of 37 studies published as of July 2021 were eligible for meta-analysis, of which 15 studies were IL-33 serum levels [11, 15, 16, 27–38] and 22 were tissue levels [10, 32, 37, 39–56]. The flowchart of the research screening process is summarized in Fig. 3. For tissue level research, all specimens are derived from cancer tissues, which were removed by biopsy or surgery, and detected by immunohistochemistry (IHC) methods. A total of 7 cancer types were treated, including gastric cancer, liver cancer, colorectal cancer, cholangiocarcinoma, gliomas, ovarian cancer, and other cancers (head and neck squamous cell carcinoma, renal cell carcinoma, squamous cell carcinoma of the tongue, non-small cell lung cancer, esophageal adenocarcinoma, and esophageal squamous cell carcinomas). Moreover, 22 clinical studies assessed IL-33 expression and correlated it with the clinicopathological features of tumors, including tumor size, histological grade, distant metastasis, lymphatic metastasis, and vascular invasion. In addition, 9 articles on the relationship between IL-33 expression and prognosis were analyzed, including 5-year OS and 3-year OS. For the study of serum levels, 6 types of cancer, including breast cancer, gastric cancer, hepatocellular carcinoma, colorectal cancer, non-small cell lung cancer and other cancers (endometrial cancer, acinar cell cancer, and nasopharyngeal carcinoma), were treated. Unfortunately, few serum-level studies have analyzed the clinicopathological features of tumors. In addition, when compiling the data, it was found that most of the included studies were Asian. The details of the included studies are shown in Tables 2 and 3.

**Table 2:** The main characteristics of a qualified study of tissue levels

Author	Year	Country	Cancer type	Antibody	Survival analysis
Quan Zhou [39]	2020	China	gastric cancer	Abcam	OS
Supaporn Yangngam [40]	2020	Thailand	cholangiocarcinoma	R&D	OS
Yan Yang [10]	2016	China	hepatocellular carcinoma	R&D	NO
Kirsten D. Mertz [41]	2015	Switzerland	colorectal cancer	R&D	NO
Zewei Wang [42]	2015	China	renal cell carcinoma	Abcam	OS
Wenwei Hu [14]	2016	China	gastric cancer	Sigma	NO
Xiaoguang Tong [44]	2015	China	epithelial ovarian cancer	Enzo Life Sciences	OS
Satoshi Nagaoka [45]	2020	Japan	cholangiocarcinoma	NA	OS
Wenxiu Wang [46]	2020	China	hepatocellular carcinoma	R&D	OS
Dorothee Grama Tzki [47]	2016	Switzerland	gliomas	R&D	OS
Jianfei Zhang [48]	2016	China	gliomas	Proteintech	OS
Yihui Wen [49]	2018	China	head and neck squamous cell carcinoma	R&D	OS
Kazuya Ishikawa [50]	2014	Japan	squamous cell carcinoma of the tongue	R&D	OS
Lu Jing [32]	2020	China	hepatocellular carcinoma	NA	NA
Chen Ling [51]	2020	China	non-small cell lung cancer	R&D	OS
Zhao Lirong [52]	2020	China	ovarian cancer	NA	NA
Xia Bingxiang [38]	2017	China	gastric cancer	Dako	OS
Zhang Jianfei [53]	2016	China	gliomas	NA	NA
Huang Di [54]	2016	China	colorectal cancer	Abcam	OS
Hu Xia [37]	2019	China	esophageal adenocarcinoma	Proteintech	NA
Yue Ying [55]	2018	China	esophageal squamous cell carcinomas	Abcam	OS
Hu Wenwei [56]	2018	China	gastric cancer	NA	NA

**Table 3:** The main characteristics of a qualified study of serum levels

Author	Year	Country	cancer type	methods	Antibody
Zhi-Ping Yang [27]	2015	Asia	breast cancer	ELISA	other
Pinghu Sun [15]	2011	Asia	gastric cancer	ELISA	R&D
Xi Zeng [16]	2016	Asia	endometrial cancer	ELISA	other
Sowa Pawel [28]	2018	Europe	acinic cell carcinoma	ELISA	R&D
Dominik Bergis [11]	2013	Europe	hepatocellular carcinoma	ELISA	R&D
Dominik Bergis [29]	2016	Europe	gastric cancer	ELISA	R&D
Jing Shen [30]	2018	Asia	hepatocellular carcinoma	Bio-Plex	other
TU Hongfei [31]	2021	Asia	gastric cancer	ELISA	made in china
Lu jing [32]	2020	Asia	hepatocellular carcinoma	ELISA	other
Shan Lingshun [33]	2020	Asia	colorectal cancer	ELISA	R&D
Niu Gang [57]	2021	Asia	gastric cancer	ELISA	made in china
Pang Pan [34]	2020	Asia	non-small-cell lung cancer	ELISA	made in china
Chang Wenlong [35]	2020	Asia	non-small-cell lung cancer	ELISA	other
Ruan Peng [58]	2019	Asia	nasopharyngeal carcinoma	ELISA	R&D
Xu Junying [36]	2016	Asia	gastric cancer	ELISA	made in china
Li Yurong [59]	2017	Asia	breast cancer	ELISA	other
Xia Bingxiang [38]	2017	Asia	gastric cancer	ELISA	made in china

### Meta-analysis results

#### Correlation of IL-33 expression with clinicopathological characteristics

##### Overall analysis

**Fig. 4:**
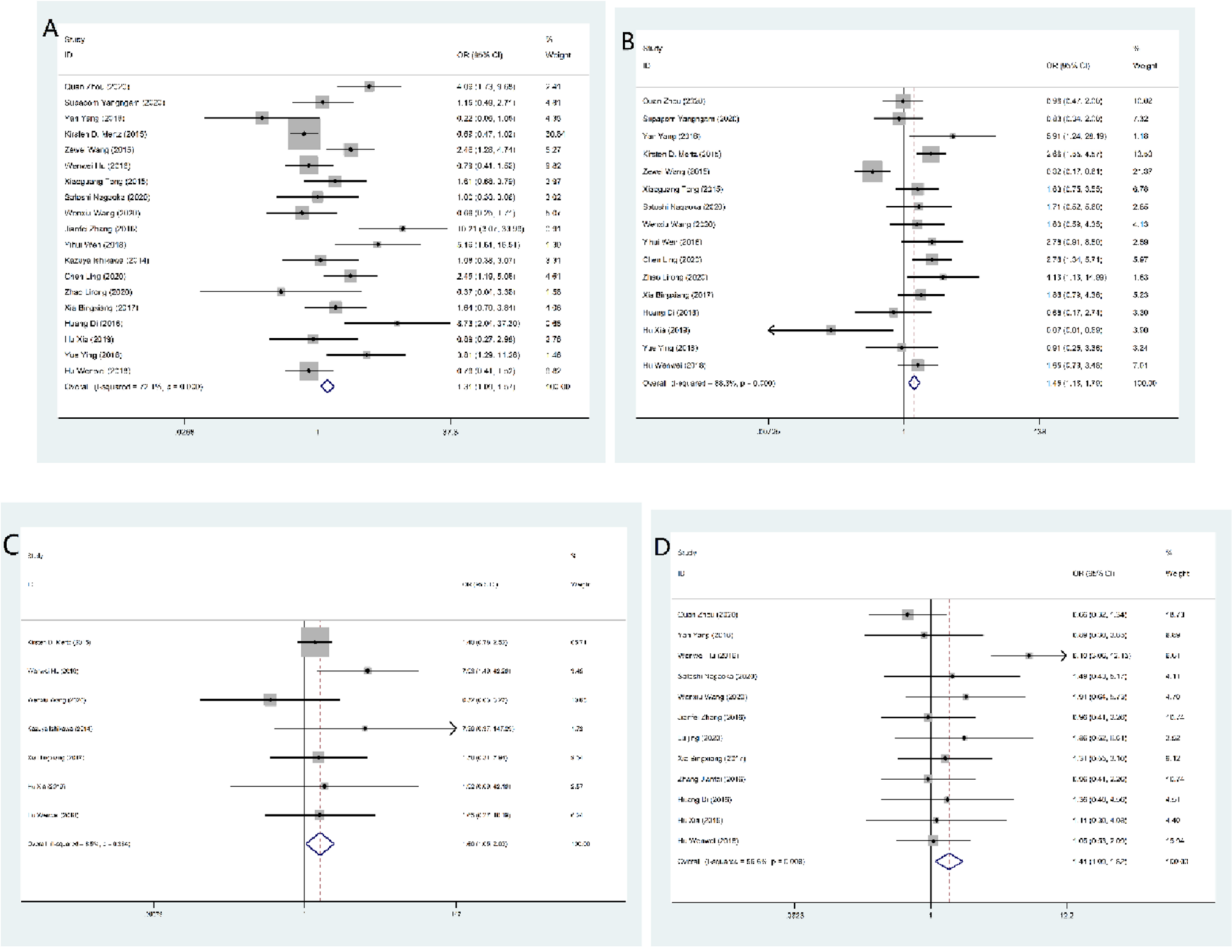
Forest plot for the association of IL-33 expression with clinicopathological parameters. High IL-33 expression was significantly associated with tumor stage (**A**), histological grade (**B**), distant metastasis (**C**), and tumor size (**D**)

Twenty-two studies investigated the relationship between IL-33 tissue levels and tumor clinicopathological parameters. Overall, the analysis showed that IL-33 expression was associated with tumor stage (OR = 1.308, 95% CI = 1.091–1.567, *p* = 0.004), histological grade (OR = 1.96, 95% CI = 1.176–1.789, *p* = 0.001), distant metastasis (OR = 1.660, 95% CI = 1.048–2.630, *p* = 0.031) and tumor size (OR = 1.406, 95% CI = 1.088–1.816, *p* = 0.009). Specifically, the high expression of IL-33 will increase the likelihood of cancer histologic grade differentiation, tumor stage, increased distant metastasis, and larger tumor size. However, no significant correlation was found for lymphatic metastasis (OR = 0.831, 95% CI = 0.642–1.075, *p* = 0.159), ductal infiltration (OR = 1.268, 95% CI = 0.885–1.815, *p* = 0.196), sex (OR = 0.867, 95% CI = 0.718–1.047, *p* = 0.138), or age (OR = 1.071, 95% CI = 0.873–1.315, *p* = 0.512). The results are summarized in Table 4 and Fig. 4.

**Table 4:** Overall analysis of the association of IL-33 expression with clinical features

Parameter	OR	95%CI	*p*	I^2^	*p*_bias_
Tumor stage	1.308	1.091–1.567	0.004	72.1%	0.053
Histological grade	1.451	1.176–1.789	0.001	68.3%	0.691
Distant metastasis	1.660	1.048–2.630	0.031	8.5%	0.392
Lymphatic invasion	0.831	0.642–1.075	0.159	56.3%	0.137
Vascular invasion	1.268	0.885–1.815	0.196	71.5%	0.883
Tumor size	1.406	1.088–1.816	0.009	56.6%	0.394
Age	1.071	0.873–1.315	0.512	14.6%	0.528
Sex	0.867	0.718–1.047	0.138	20.2%	0.193
3-year OS rate	3.467	2.563–4.690	< 0.000	0	
5-year OS rate	2.784	2.001–3.873	< 0.000	19.7%

##### Subcategory analysis

We used subgroup analysis to explore potential sources of heterogeneity, including patient region and antibodies used in the study. The main clinicopathologic parameters, including tumor stage, histological grade, lymphatic invasion, vascular invasion, and tumor size, were discussed. It was statistically found that in the subgroup analysis of the patient's area, the expression of IL-33 protein in the Asian group was found to be positively correlated with tumor stage (OR = 1.582, 95% CI = 1.285–1.946, *p* < 0.000), tumor size (OR = 1.184, 95% CI = 1.044–1.343, *p* = 0.008) and histological grade (OR = 1.262, 95% CI = 1.001–1.591,* p* = 0.049), which was consistent with the overall analysis. In the European subgroup, IL-33 expression was positively correlated with histological grade (OR = 2.660, 95% CI = 1.550–4.566, *p* < 0.000). In addition, IL-33 expression was not associated with distant metastasis, lymphatic metastasis, or vascular invasion regardless of patient location (*p* > 0.05). For the analysis of antibody subgroups, the results of different subtypes were different. In the R&D group, lymphatic metastasis (OR = 0.631, 95% CI = 0.413–0.964, *p* = 0.033) and histological grade (OR = 2.284, 95% CI = 1.642–3.177, *p* < 0.000) were positively correlated with IL-33 expression. In the Abcam group, the expression of IL-33 was positively correlated with tumor stage (OR = 3.478, 95% CI = 2.237–5.407, *p* < 0.000) and negatively correlated with histological grade (OR = 0.571, 95% CI = 0.372–0.877), *p* = 0.011) but was not correlated with tumor size or lymphatic metastasis. In the Sigma group, only tumor size was positively correlated with IL-33 expression (OR = 1.878, 95% CI = 1.367–2.581, *p* < 0.000). In the Proteintech group, IL-33 expression was positively correlated with tumor stage (OR = 3.206, 95% CI = 1.511–6.802, *p* = 0.002). In the other group, histological grade IL-33 expression was positively correlated with histological grade (OR = 1.614, 95% CI = 1.097–2.375, *p* = 0.015). The results are summarized in Tables 5 and 6.

**Table 5:** Main stratified analysis results of meta-analysis of IL-33 expression (Antibody part)

**Variables**	R&D			**Abcam**			**Sigma**			**Proteintech**			**other**		
	**OR(95% CI)**	**p**^**a**^	**I**^**2**^	**OR(95% CI)**	**p**^**a**^	**I**^**2**^**(%)**	**OR(95% CI)**	**p**^**a**^	**I**^**2**^**(%)**	**OR(95% CI)**	**p**^**a**^	**I**^**2**^**(%**	**OR(95% CI)**	**p**^**a**^	**I**^**2**^**(%)**
Tumor stage	**0.973** **(0.746 -1.268)**	**0.838**	**72.1**	**3.478** **(2.237–5.407)**	**0.000**	**0.0**	**0.792** **(0.499–1.257)**	**0.323**	**0**	**3.206(1.511 -6.802)**	**0.002**	**87.3**	**1.317** **(0.793–2.188)**	**0.287**	**0.0**
Histological grade	**2.284** (1.642–3.177) *	**0.000**	**34.8**	**0.571** **(0.372–0.877)**	**0.011**	**41.5**							**1.614** **(1.097–2.375)**	**0.015**	**46.9**
Distant metastasis	**0.722** **(0.419–1.244)**	**0.240**	**26.3**				**0.257** **(0.081–0.820)**	**0.022**	**35.3**				**0.606** **(0.145–2.529)**	**0.912**	**0.0**
Lymphatic invasion	**0.631** **(0.413–0.964)**	**0.033**	**66.8**	**0.833** **(0.484–1.435)**	**0.510**	**71.7**	**1.504** **(0.912–2.479)**	**0**	**0.109**				**0.562** **(0.283–1.116)**	**0.100**	**0**
Vascular invasion	**0.734** **(0.459–1.173)**	**0.196**	**69.9**										**2.787** **(1.577–4.926)**	**0**	**0**
Tumor size	**1.156** **(0.764–1.750)**	**0.493**	**25.5**	**0.963** **(0.717–1.293)**	**0.801**	**18.9**	**1.878** **(1.367–2.581)**	**0.000**	**91.4**	**1.001** **(0.781–1.284)**	**0.992**	**0**	**1.066** **(0.867–1.311)**	**0.544**	**0**
3-year OS rate	**3.511** **(2.227–5.536)**	** < 0.000**	**0**	**4.972** **(2.116–11.687)**	** < 0.000**	**23.5**							**2.849** **(1.666–4.871)**	** < 0.000**	**0**
5-year OS rate	**3.902** **(2.086 -7.298)**	** < 0.000**	**0**	**3.002** **(1.565- 5.756)**	**0.001**	**17.6**							**2.089** **(1.270 -3.437)**	**0.004**	**0**

**Table 6:** Main stratified analysis results of the meta-analysis of IL-33 expression (regional distribution)

	Asia			Europe		
Parameter	OR	95%CI	*p*	OR	95%CI	*p*
Tumor stage	1.582	1.285–1.946	< 0.000	0.694	0.472–1.019	0.063
Histological grade	1.262	1.001–1.591	0.049	2.660	1.550 -4.566	< 0.000
Distant metastasis	0.462	0.215–1.002	0.051	1.615	0.613–4.251	0.261
Lymphatic invasion	0.782	0.597–1.026	0.076	1.615	0.613 -4.251	0.332
Vascular invasion	1.244	0.797–1.940	0.336	1.311	0.712 -2.416	0.385
Tumor size	1.184	1.044–1.343	0.008			
Age	1.067	0.868–1.312	0.539	1.333	0.283–6.279	0.716
Sex	0.895	0.740–1.082	0.503	0.051	0.005 -0.514	0.012
3-year OS rate	3.467	2.563–4.690	< 0.000			
5-year OS rate	2.784	2.001–3.873	< 0.000			

#### Impact of IL-33 expression on survival for cancer patients

A total of 9 studies were included in the analysis to study the relationship between IL-33 expression and the prognosis of cancer patients. The standard prognostic indicators are 5-year OS and 3-year OS.

##### Overall analysis

**Fig. 5:**
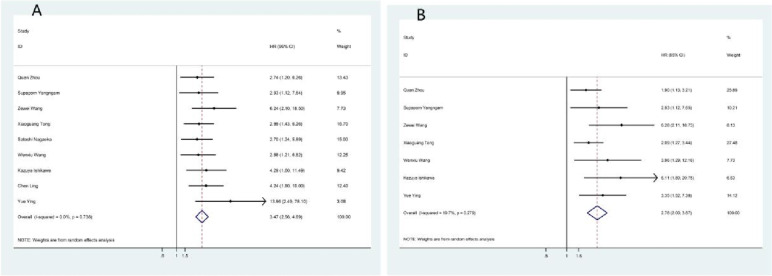
Meta-analysis of the association between IL-33 expression and prognosis indicators by 3-year OS (**A**) and 5-year OS (**B**)

Data from this analysis indicated that cancer patients with high IL-33 expression had a poorer prognosis. Patients with higher IL-33 expression showed poor 3-year OS (OR = 3.467, 95% CI = 2.563–4.690, *p* < 0.000) and 5-year OS (OR = 2.784, 95% CI = 2.001–3.873, *p* < 0.000), as shown in Fig. 5 and Table 4.

#### Correlation of serum IL-33 expression with cancer patients

**Fig. 6:**
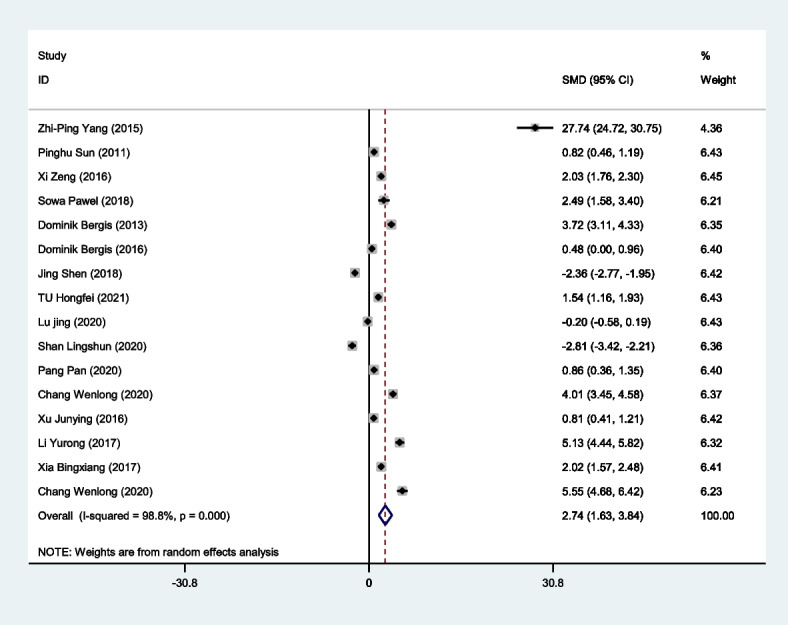
Forest plot for the correlation of serum IL-33 expression with cancer patients by meta-analysis

The pooled SMD showed that compared with the control group, the serum IL-33 level of the cancer group was significantly higher (SMD = 2.847, 95% CI = 1.766–3.929, *p* < 0.000), as shown in Table 7 and Fig. 6. Significant heterogeneity was observed (I^2^ = 98.8%; *p* < 0.000). To reduce the heterogeneity of the included studies, we compared the serum IL-33 levels of different cancer type subgroups and control groups. The serum IL-33 level was higher in the gastric cancer group (SMD = 1.770, 95% CI = 0.904–2.637, *p* < 0.000). In the subgroup analysis of the patient's region, we found that the serum IL-33 level was higher in the Asian group (SMD = 3.011, 95% CI = 1.774–4.249, *p* < 0.000). Among the antibody types, in the Made in China group (SMD = 2.025, 95% CI = 1.031–3.080, *P* < 0.000) and the other group (SMD = 5.368, 95% CI = 2.767–7.969, *p* < 0.000), serum IL-33 expression increased. In addition, we also discussed the relationship between IL-33 serum expression and major clinicopathological parameters, including tumor stage, lymphatic invasion, vascular invasion, and tumor size. The results showed that the serum level of IL-33 was positively correlated with tumor stage (SMD = 0.936, 95% CI = 0.555–1.317, *p* < 0.000) and vascular invasion (SMD = 0.601, 95% CI = 0.387–0.815, *p* < 0.000), which was consistent with the results of the IL-33 tissue level. The results are summarized in Tables 7 and 8.

**Table 7:** Results of meta-analysis for IL-33 and cancer risk

Parameter	SMD	95%CI	*p*	I^2^
Total	2.735	1.632–3.838	< 0.000	98.8
Breast cancer	16.385	-5.766–38.536	0.147	99.5
Gastric cancer	1.138	0.620–1.656	< 0.000	87.3
Hepatocellular carcinoma	0.379	-2.654–3.412	0.807	99.2
Non-small-cell lung cancer	3.457	0.738–6.157	0.013	98.3
Other cansers	0.566	-2.740–3.872	0.265	99.1
Ethnicities				
Asia	2.886	1.616–4.155	< 0.000	99.0
Europe	2.223	0.049–4.397	0.045	97.1
Antibody				
R&D	0.932	-0.958–2.823	0.334	98.3
Made in china	1.311	0.755–1.866	< 0.000	85.0
Other	5.368	2.767–7.969	< 0.000	99.4

**Table 8:** Main stratified analysis results of the meta-analysis of IL-33 expression

Parameter	SMD	95%CI	*p*	I^2^(%)
Tumor stage	0.936	0.555–1.317	< 0.000	69.3
Lymphatic invasion	0.594	0.056–1.133	0.031	82.2
Vascular invasion	0.601	0.387–0.815	< 0.000	0
Tumor size	0.447	-1.245–2.139	0.605	96.8
Age	0.153	-0.101–0.406	0.238	48.0
Sex	-0.101	-0.323–0.120	0.368	18.1

#### Sensitivity analysis and publication bias

**Fig. 7:**
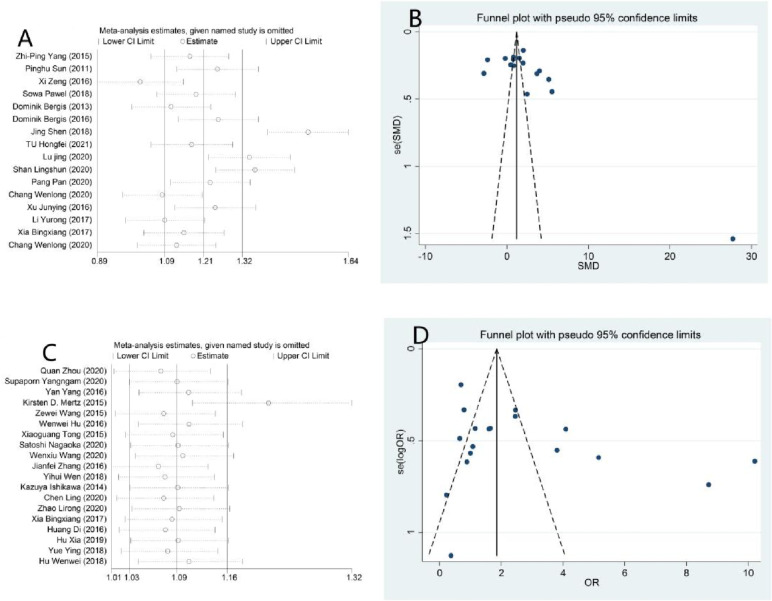
Funnel plot of publication bias for evaluating the association of IL-33 expression with clinicopathological parameters of cancer by sensitivity analysis of serum (**A**), funnel plot of serum (**B**), sensitivity analysis of protein (**C**) and funnel plot of protein (**D**)

To detect the impact of a single paper on the entire dataset, we conducted a sensitivity analysis. The results in Table 2 show that none of the studies substantially changed the results, which indicated that the results of our meta-analysis were statistically stable. In addition, we used Egger’s test and Begg’s funnel plot to assess publication bias (Fig. 7). Neither Egger’s test nor Begg’s funnel plot showed any publication bias in the expression of IL-33 (*p* > 0.05). (IL-33 serum level expression: Begg’s test: *p* = 0.063; Egger’s test: *p* = 0.065). Therefore, our results were credible because there was no significant publication bias in our meta-analysis.

## Discussion

Building evidence shos that IL-33 plays a key role in several tumorigeneses by affecting tumor stem cells, tumor growth, metastasis, angiogenesis, and other many other tumorigenesis factors. [13, 60]. In comparison to the healthy control group, HCC patients demonstrated elevated levels of ST2 mRNA and serum in their peripheral blood. However, no significant differences were found in IL-33 mRNA and protein levels between HCC patients and controls. Nevertheless, the findings regarding IL-33 and ST2 levels in serum were consistent with the results obtained through RT-PCR, which align with the findings of Wei et al. [61] and the results obtained in our meta-analysis. Namely, in the subgroup analysis of cancer types in the meta-analysis, it was found that serum IL-33 expression had no effect on the occurrence and progression of HCC, which may be due to the inclusion of only 3 studies. However, IL-33 is a dual-function cytokine, and the expression data in different cancer types are inconsistent. Even in the same cancer type of HCC, inconsistent results have been observed. Some studies have found that the serum IL-33 level of patients with HCC is higher than that of normal people [8]. However, Bergis et al. did not find a significant difference in serum levels of IL-33 between patients and healthy controls [44], which is consistent with our findings.

In our meta-analysis, we found that IL-33 is abnormally expressed in a variety of tumors (such as lung cancer, breast cancer, colorectal cancer, etc.), participates in the occurrence, development and metastasis of tumors and even plays a dual role in promoting tumors and antitumor effects within the same tumor type [13, 62–67]. Although there have been many studies, including mechanistic studies and clinical studies, the role of IL-33 in tumors is still controversial. Therefore, we screened 37 studies that met the inclusion criteria from the tissue level and serum level of IL-33 to conduct a relatively comprehensive meta-analysis. The results showed that IL-33 was a poor predictor in cancer patients. IL-33 overexpression was positively correlated with tumor stage, histological grade, distant metastasis and tumor size, and the expression of IL-33 was correlated with a low 5-year OS rate and 3-year OS rate in cancer patients.

In the IL-33 tissue level section, 22 studies were included in the analysis. We first studied the correlation between IL-33 expression and clinicopathological characteristics. The results showed that the high expression of IL-33 was positively correlated with tumor stage, histological grade, distant metastasis, and tumor size. Specifically, the high expression of IL-33 would increase the histological grade differentiation of cancer, increase the possibility of distant metastasis, and make tumors larger in size and later stage. Due to the large heterogeneity in some groups, we explored potential sources of heterogeneity by subclass analysis based on patient regions and antibodies used in the study. Statistically, it was found that the expression of IL-33 was positively correlated with the tumor stage, tumor size and histological grade of the Asian subgroup in the subgroup analysis of the patient's location, which was consistent with the results of the overall analysis. In the European subgroup, IL-33 expression was positively correlated with histological grade. In addition, regardless of where the patient was located, IL-33 expression had nothing to do with distant metastasis, lymphatic invasion, vascular invasion, age and sex, which may be due to insufficient literature meeting the inclusion criteria. For subclass analysis of antibodies, the results varied among subgroups. In the R&D group, the expression of IL-33 was negatively correlated with lymphatic invasion and positively correlated with histological grade. In the Abcam group, IL-33 expression was positively correlated with tumor stage and negatively correlated with histological grade but not with tumor size or lymphatic invasion. In the Sigma group, only tumor size was positively correlated with IL-33 expression. In the Proteintech group, IL-33 expression was positively correlated with tumor stage. This shows us the direction of choosing antibodies when we detect IL-33 expression according to the purpose of detection. However, in the subgroup analysis, we only collected data from the Asian population in the regional distribution of patients. This may not fully account for genetic and environmental differences between ethnic groups.

In the IL-33 serum expression part, the expression level of IL-33 in tumor patients was higher than that in the control group, and the expression level of IL-33 serum was positively correlated with tumor stage and vascular invasion, which was consistent with the result of the IL-33 tissue level. In the analysis of cancer type subgroups, it was found that the serum levels of IL-33 in the gastric cancer group and non-small cell lung cancer group were higher than those in the control group. Therefore, IL-33 has clinical value as a potential diagnostic tool for tumor patients, especially for gastric cancer and non-small cell lung cancer. Significant heterogeneity was observed in the study. This heterogeneity persisted after the subgroup analysis, suggesting that the study population, measuring reagents, and other covariables may account for the difference. In addition, we did not obtain any data from studies in America, Africa and Oceania, and a series of factors that affect the serum levels of IL-33 in cancer patients, including age, sex, and disease progression, could cause heterogeneity. However, the association between serum IL-33 expression level and survival was not analyzed due to the few studies meeting the inclusion criteria.

The IL-33 signaling pathway is mediated through its receptor ST2, which, upon binding to ST2, results in nuclear signaling and immunomodulatory action in various cells (tumors, immune, heart). However, there are limited data in tumors. Studies have found that the ST2 level was higher in tumor patients than in healthy people (1079. 6 ± 310.1 vs. 218.6 ± 45. 8, *p* < 0.05). ST2 was associated with advanced and metastatic disease in gastric cancer patients and significantly correlated with the duration of the disease [29]. In addition, Quan Zhou et al. found that ST2 was upregulated in human gastric cancer and served as a prognostic marker for poor survival of gastric cancer patients [39]. Unfortunately, there were few studies describing the relationship between serum and tissue level levels of ST2 and tumors. Therefore, the association between ST2 levels and tumors was not included in this meta-analysis.

Although we conducted a detailed meta-analysis, there were still some potential limitations. First, we evaluated the 5-year survival rate and the 3-year survival rate from the Kaplan‒Meier curve. These estimated data may not be as reliable as the direct data of the original study, and the number of included research articles was relatively small. Second, although the random effects model, subclass analysis and sensitivity analysis were applied to correct the heterogeneity, there was large heterogeneity in some subgroups. Third, while we evaluated publication bias and found no significant bias, it is worth noting that papers with positive results tend to be published. Therefore, the correlation between IL-33 expression and the prognosis of tumor patients may exceed our calculation. In addition, due to limited specimen collection, we only detected the expression level of IL-33 in the serum of patients with HCC but not in patients with other tumors.

In conclusion, despite some limitations, the results of this study suggest that high IL-33 expression was positively associated with higher tumor stage, lymphatic invasion, and other pathological characteristics. In addition, high IL-33 expression was associated with poor prognosis in cancer patients. Moreover, comprehensive analysis of the expression of IL-33 in tumors can not only help us better understand the role of IL-33 in tumorigenesis but also find that it may be a useful predictive or diagnostic biomarker for tumors. However, larger prospective studies may be needed in the future to verify our results.

## Data Availability

The datasets used and analyzed during the current study are available from the corresponding author on reasonable request.
